# Editorial: New perspectives on procrastination, volume II

**DOI:** 10.3389/fpsyg.2022.994413

**Published:** 2022-08-03

**Authors:** Piers Steel, Frode Svartdal

**Affiliations:** ^1^Haskayne School of Business, University of Calgary, Calgary, AB, Canada; ^2^Department of Psychology, UiT the Arctic University of Norway, Tromsø, Norway

**Keywords:** procrastination, review, assessment, multi-level modeling, comparative psychology

The field of procrastination is growing almost exponentially, with the academic search engine *Semantic Scholar* indicating approximately half of the more than 16,000 articles written on the topic were published in the previous 6 years. At this point, review articles of the field are becoming increasingly important. In addition, foundational issues still are being addressed, in particular the diagnosis and assessment of procrastination and its effects. More sophisticated techniques are being increasingly employed, with hierarchical or multi-level modeling proving particularly useful. Finally, a mature science increasingly moves into intervention or treatment, building on the previous components. Each of these components are featured in this Research Topic.

As a review of procrastination, Yan and Zhang conducted bibliometric analysis of the field based on 1,635 articles. It is a useful examination of where the research has been as well as where it may be going. Those looking to familiarize with the overall field will find this invaluable. For example, as they found and as reflected here, bedtime procrastination is an emerging focus. Also, they note that there have been considerable challenges in studying the topic, with our preponderance of using student samples and self-reports, issues that notably some of the very articles we feature here have overcome. Along these lines, Zentall used comparative psychology to connect procrastination to basic behavioral processes, indicating that though culture, skills and tasks characteristics can exacerbate procrastination, it is tapping into fundamental aspects of our cognitive architecture. Consistent with this conclusion, Lu et al. established meta-analytically that procrastination is common across a wide range of sociodemographic situations and characteristics.

Foundational to any field is its assessment, with Vangsness et al.'s examination of ten popular scales finding various problems, with only the *Irrational Procrastination Scale* demonstrating both a consistent factor structure and a strong predictor of behavioral delay. Svartdal and Nemtcan found that further improvements can be made in identifying the most dysfunctional forms of procrastination (i.e., differentiating among strategic, inconsequential and irrational delays) by focusing the past negative consequences of unnecessary delay. Across two studies, they developed the *Negative Consequences of Procrastination* (NCP) scale, which pairs well with Rozental et al.'s research. They sought to identify those most need of support, finding that *Pathological Delay Criteria*, which is analogous to past negative consequences, an excellent indicator. Together, these two assessments clearly help to pinpoint those in need of clinical care.

Two papers address the relationship between procrastination and sleep. Meng et al. focused on the antecedents of bedtime procrastination, demonstrating the negative role of low self-efficacy. Furthermore, they explored the roles of moderators of this effect, finding a significant moderating effect of negative affect. Maier et al. explored the consequences insufficient sleep has for procrastination in full-time employees. Using a multilevel design with repeated measures, they demonstrated a negative relation between sleep quality in a given night and procrastination the following day. Importantly, this effect was demonstrated to be stronger for later chronotypes (i.e., evening types).

The use of sophisticated designs in the analysis of antecedents and outcomes of procrastination was further illustrated in the paper by Kljajic et al. Employing a multilevel design, they compared students to one another (between-person level) as well as students over different courses (within-person level). At both levels, they explored the mediating role of procrastination in the associations between two antecedents (autonomous and controlled motivation) and two outcomes (grade and wellbeing). In their case, a main finding was that the antecedent of procrastination differed across levels of analysis. Also using multilevel modeling, Steel et al. examined the causes and impact of procrastination on “slippery deadlines” (i.e., deadlines where the due date is ill-defined and can be autonomously extended). Performance data from a unique venue, Canadian arbitration cases, as well as associated survey data allowed for analyses in terms of individual differences, self-regulatory skills, workloads, and task characteristics on the outcome variable, time delay. The results of this study, as well as in a replication using an independent data set, indicated trait procrastination to be a substantive predictor of observed delay. Analyzing academic procrastination, Wieland et al. applied an event-based experience sampling method to assess the momentary appraisals of the tasks at hand as well as their next day intention to work on the tasks. In addition, a second query administered the following day repeated the appraisal queries as well as probed whether the intentions the previous day had been implemented or delayed. A devaluation of the study-related tasks increased the risk for an actual delay, and a measure of general procrastination tendency did not predict individual differences in their task-specific delay behavior. These three papers all demonstrate multilevel modeling as a welcome addition to cross-sectional surveys.

Finally, Schunemann et al. describe a promising approach to overcome procrastination. Consistent with previous studies that have indicated that procrastination is rooted in use of dysfunctional emotion regulation strategies, particularly in dealing with negative emotions, the authors argue that training of adaptive emotion regulation skills should help alleviate procrastination. Using a randomized controlled design, the intervention group received an online emotion regulation training over a period of 9 weeks. Results demonstrated that the enhancement of general emotion regulation skills significantly reduced subsequent procrastination behavior, and subsequent mediation analyses indicated that the reduction of procrastination was significantly mediated by the increase in general emotional regulation skills.

Altogether, these papers indicate that the study of procrastination is continuing to mature. A better measurement base is being established as well as a deeper understanding of the underlying mechanisms. Research designs are becoming more sophisticated and there are increasing signs of the most important indicator of maturity: effective interventions. At this point, we believe the field is ready to tackle enthusiastically how to treat or diminish procrastination. We have multiple venues where procrastination is rampant and participants are plentiful, particularly Massive Open Online Courses (MOOCs).

Looking forward, we expect that there will be interactions between people and treatment, meaning that one size does not fit all. Procrastinators share common characteristics (e.g., impulsiveness), but relevant individual differences should be considered. Furthermore, diagnostics and interventions should assess not only degree and causes of procrastination but also the presence or absence of compensating self-regulatory skills as this will help to adeptly match individuals with the myriad of possible treatments (e.g., if someone has already mastered a skill, further training is unlikely to be beneficial). Even situational characteristics should be integrated in the understanding of procrastination, as some environments (e.g., the academic) tolerate procrastination more than others. We will need to establish what interventions pair well together, what is sustainable and people's readiness to adopt them. This can touch on everything from work design to recuperation. It appears that procrastination and our study of it will continue to grow for quite a while.

## Author contributions

All authors listed have made a substantial, direct, and intellectual contribution to the work and approved it for publication.

## Conflict of interest

The authors declare that the research was conducted in the absence of any commercial or financial relationships that could be construed as a potential conflict of interest.

## Publisher's note

All claims expressed in this article are solely those of the authors and do not necessarily represent those of their affiliated organizations, or those of the publisher, the editors and the reviewers. Any product that may be evaluated in this article, or claim that may be made by its manufacturer, is not guaranteed or endorsed by the publisher.

